# Frequency and types of pre-analytical errors in a clinical laboratory of a specialized healthcare hospital

**DOI:** 10.12669/pjms.40.2(ICON).8963

**Published:** 2024-01

**Authors:** Aroofa Tasneem, Muhammad Zubair, Zoha Rasool, Farrukh Zia Tareen

**Affiliations:** 1 Aroofa Tasneem, MBBS, Resident MCPS. Clinical Pathology, Multan Institute of Kidney Diseases, Multan, Pakistan; 2 Muhammad Zubair, MBBS, FCPS Consultant Chemical Pathologist, Head of Laboratories, Multan Institute of Kidney Diseases, Multan, Pakistan; 3 Zoha Rasool, MBBS, Resident MCPS, Clinical Pathology, Multan Institute of Kidney Diseases, Multan, Pakistan; 4 Farrukh Zia Tareen, MBBS, FCPS, Consultant Hematologist, Multan Institute of Kidney Diseases, Multan, Pakistan

**Keywords:** Clinical laboratory, Specimens, Pre-analytical errors

## Abstract

**Background & Objective:**

The pre-analytical phase, encompassing all preparatory steps leading to the analytical process, represents a critical stage prone to laboratory errors. An assessment of the occurrence and categories of laboratory errors, specifically within the pre-analytical phase of laboratory procedures, can guide in taking timely actions for rectifying errors responsible for damage and loss of samples. This study aimed to assess the frequency and types of pre-analytical errors within a clinical laboratory at the Multan Institute of Kidney Diseases over two years.

**Methods:**

This research took place at the Multan Institute of Kidney Diseases. Data was extracted from the hospital laboratory records of the period from 1^st^ January 2021 to 31^st^ December 2022. After data compilation, a retrospective cross-sectional methodology was adopted to assess frequency and types of pre-analytical errors within a clinical laboratory. The records underwent a thorough examination to identify pre-analytical errors, which were classified according to their type and occurrence rate.

**Results:**

Among the 254810 specimens received during the data collection period, a total of 1,722 specimens (0.67% of all collected samples) were found unsuitable for further processing. Amongst the rejected specimens, 718 (41.6%) displayed indications of hemolysis, 388 (22.5%) exhibited clotting, 217 (12.6%) had an insufficient volume and the remaining specimens fell into other miscellaneous categories such as insufficient quantity, unlabeled samples etc.

**Conclusion:**

The overall percentage of sample rejections in the laboratory was 0.67%. This study provided valuable insights into various reasons, and causes that require improvements to enhance the efficiency and quality of laboratory processes.

## INTRODUCTION

In today’s world, healthcare laboratories are crucial for providing accurate information for clinical decision-making.^1^ Despite the presence of advanced automation in diagnostic laboratories, significant error rates persist within clinical laboratories.^1^ It is essential to comprehend and acknowledge the origins of these errors to effectively address unexpected laboratory results which do not align with clinical information.^2^ A myriad of causes may exist. Sometimes, even, the specific circumstances at the regional level, such as pandemics, possess the capacity to amplify the rate of pre-analytical errors.^3^

**Fig.1:**
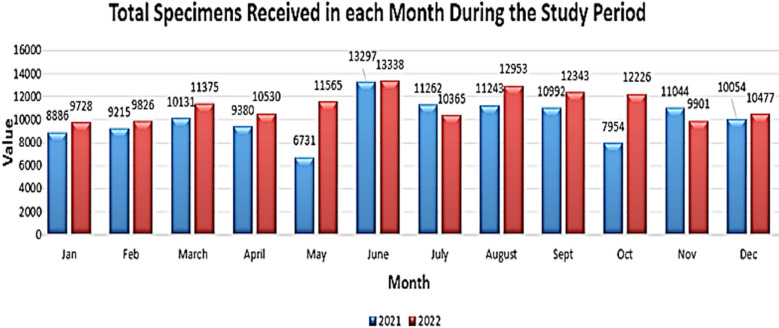
Total Specimens Received in Two Years.

Lab testing commences with requisitions entered by consultants and medical officers from different departments of the hospital. Following this, blood samples are collected by phlebotomists or nursing staff and transported to the lab in temperature-controlled boxes. A lab technologist then examines the samples for pre-analytical errors, requesting fresh samples if any issues are identified. Over the study duration, a total of 254,816 specimens were received from various departments, utilizing vacuum tubes for collection, and pre-analytical error categories were established. The accompanying graph (Fig.1) illustrates the total number of specimens received during this period.

In short, it encompasses ordering, specimen collection, transportation, preparation, analysis, and result reporting.^4^ Laboratory errors can be categorized into three distinct subcategories: pre-analytical, analytical, and post-analytical errors. Pre-analytical errors, the most common type, happen before analysis and result from mistakes in specimen handling and transportation. Analytical errors occur during analysis but have decreased due to improved standardization and quality control. Post-analytical errors occur during result transmission and interpretation.^4^

ISO 15189:2012 defines the pre-analytical phase as per its guidelines. “Steps starting in chronological request, from the clinician’s request and including the examination requisition, preparation of the patient, collection of the primary sample, and transportation to and within the laboratory, and ending when the analytical examination procedure begins”.^5^

Pre-analytical errors contribute to a substantial proportion of laboratory errors, accounting for roughly 70% of the total laboratory errors.^6^ Thus, the rationale of this study rested in addressing the critical pre-analytical phase of laboratory procedures, which are prone to a most significant number of errors, emphasizing the need to know their ratios and mitigate them. Before this study, there was a lack of data and a knowledge gap on this topic, especially in South Punjab, a backward region with a low literacy rate. Results of this study can profoundly impact the precision and trustworthiness of laboratory findings resulting in incorrect diagnoses, inappropriate medical interventions, and higher healthcare expenses.

To address this issue, staff training is also essential in reducing errors across different situations. It is essential to consider both the knowledge retained by trainees after completing their training and the ongoing training process for continuous improvement.^7^ Furthermore, utilization of training interventions can lead to cost savings by effectively reducing pre-analytical errors.^7^-^10^ In short, a highly efficient approach to reducing the risk of pre-analytical errors involves implementing effective training and relying on well-established quality indicators known for their effectiveness in monitoring and process improvement.^11^,^12^

## METHODS

A cross-sectional retrospective study was conducted at Multan Institute of Kidney Diseases, Multan. It spanned over a duration of two years, from January 2021 to December 2022 using a retrospective, cross-sectional study design.

### Inclusion & Exclusion criteria

All types of specimens, whether, blood or body fluid (pus, sputum, urine, high vaginal swabs, pleural and peritoneal fluid, etc.) received (whether accepted or rejected during study period) were included while none of the stored specimens were excluded.

### Ethics Committee approval

The author received official approval from the institutional review board under IRB Number: IHHN_IRB_2023_06_008.

### Data collection procedure

The lab registers having the total specimen received and rejected data were viewed. This data was compiled in Excel spreadsheets according to various criteria such as source of specimen, rejection for reason or responsible personnel. Statistical analysis was done. The following bars show number of each specimen received during the period.

### Statistical analysis

Microsoft Excel was utilized for bar graphs and Python’s SciPy for robust statistical analysis, employing the chi-squared test to assess variable relationships through observed and expected frequencies in different categories. This integrated approach enhanced our comprehensive dataset understanding with precise analysis and meaningful visualization.

## RESULTS

**Fig.2:**
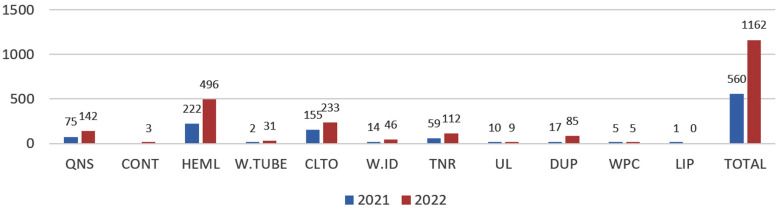
Total number of rejected specimens in 2021 & 2022 according to various codes.

Out of the total samples, 1722 were found to have at least one pre-analytical error. The accompanying tables and graphs depict details of the rejection based on their various location, departments, responsible personnel etc. Table-I and II. Fig.1 and Fig.2.

**Table-I:** Contingency Table to Perform Chi-Squared Test for Independence.

	2021	2022
Errors	726	996
No Errors	119,463	133,631

**Table-II:** Compare Pre-Analytical Errors between Nurses and Phlebotomists

Year	Healthcare Professional	Total Specimens	Pre-analytical Errors	Pre-analytical Error Frequency (%)
2021	Nurses	61399	668	1.08%
2021	Phlebotomists	58790	58	0.09%
2022	Nurses	68142	914	1.34%
2022	Phlebotomists	66485	82	0.12%

**Table-III:** Year-wise Comparison of Data and Report Frequencies.

Year	Total Specimens	Total Pre-analytical Errors	Pre-analytical Error Frequency (%)	P-value
2021	120189	726	0.60%	0.88
2022	134627	996	0.73%	

**Fig.3:**
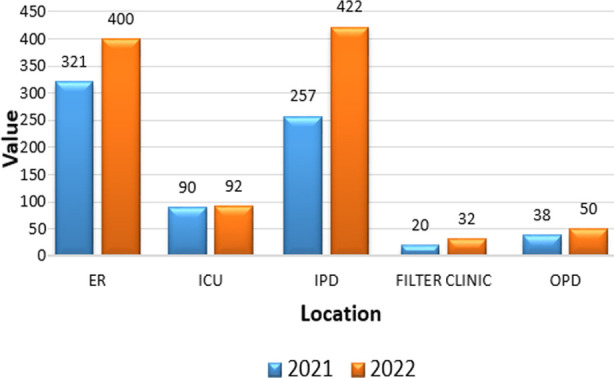
Number of rejected specimens received from various departments in the study period.

The chi-squared test for independence was conducted to compare the proportions of pre-analytical errors between the years 2021 and 2022. The calculated chi-squared statistic was approximately 0.0216 with one degree of freedom. The p-value associated with this statistic was approximately 0.8832.

## DISCUSSION

Our two-year retrospective study highlights the frequency and types of pre-analytical variables that resulted in sample rejection. There were a total of 1722 unsuitable specimens and the majority of blood samples were rejected owing to hemolysis. Several factors contribute to specimen hemolysis, including forcing blood through a fine needle, vigorous tube shaking, and premature centrifugation before clotting. It’s important to note that red top tubes without preservatives should not be shaken after sample collection, while plasma tubes should be gently inverted to ensure proper mixing of the anticoagulant with the blood.

Haroon and his coworkers found faulty techniques among phlebotomists as the main culprit for pre-analytical error.^13^ The main cause of hemolysis was wrong technique and our results were comparable with their findings. We found clotting to be a second major culprit. Clot formation primarily occurs due to the improper mixing of anticoagulated tubes, leading to inaccurate results, especially in coagulation profiles. Even small clots can falsely prolong prothrombin time. This process damages cells and depletes coagulation factors, making the sample unsuitable for assays requiring plasma or whole blood. In contrast to ours, Singh et al. found clotting as the most frequent error in the pre-analytical process.^14^

We found a higher rejection rate among specimens received from critical patients in the ER, ICU, and IPD. This finding is backed by a study by Keskin & co. where various factors, such as prolonged bed rest, critical emergencies, underlying illnesses, ongoing treatments, or frequent blood draws, can contribute to pre-analytical rejection rates.^15^

Finding the different ways and solutions to prevent errors from occurring in the pre-analytical phase is important. Abbas et al. found that total lab automation has the potential to reduce pre-analytical errors and improve efficiency.^16^ We also found out that there are higher rejection rates among specimens drawn by nurses than phlebotomists. This can be attributed to lack of awareness about good sampling practices and lagging behind phlebotomy specific knowledge on part of nurses. A study assessed the awareness of pre-analytical errors among primary phlebotomist nurses. Diploma-qualified nurses demonstrated higher overall awareness, but their understanding of recommended sampling practices was inadequate. To enhance accuracy in laboratory testing, targeted educational interventions can be one of the solutions to improve knowledge, promote best practices, and prevent errors in the future.^17^

Naz S et al. has suggested that laboratory staff should embrace a comprehensive perspective toward laboratory diagnosis and work closely with clinicians to deliver effective diagnostic services to patients. Ensuring the adoption of quality control, along with regular appraisals and audits, is imperative to protect patient interests and provide high-quality services.^18^

Quality improvement activities have been recognized as effective strategies to reduce pre-analytical errors. Lee conducted a study at the University Hospital of Korea, demonstrating the positive influence of quality improvement activities on minimizing pre-analytical errors in clinical laboratories.^19^

The above discussion reveals that pre-analytical errors are primarily attributed to improper phlebotomy practices, which may result from factors such as lack of awareness or high workload. It is imperative to take action in promoting optimal phlebotomy practices among healthcare professionals.^20^,^21^ Errors can also arise due to non-specialized personnel and a lack of clarity regarding standardized methods and transportation durations for various tests. To address this issue, it is crucial to provide proper training regarding the proper collection and handling of blood samples for the healthcare professionals involved. The use of specialized vessels and ensuring prompt transportation of samples by experienced individuals can also help minimize errors and improve patient care.

### Strength of study

Our study will help identifying weaknesses, improving quality, and aiming to enhance patient safety, cost-effectiveness, workflow efficiency, standardization, informed decision-making, continuous advancement, educational initiatives, and positive clinical outcomes in the future.

### Limitations of the study

Since the data collection was limited to a single specialized healthcare unit in Multan, the generalizability of this study is restricted, and more research is needed to uncover the underlying causes of these pre-analytical errors.

## CONCLUSION

Despite advancements in the field of pathology, persistent pre-analytical errors stem from human intervention. Effective solutions involve competency checks, training, standardization, collaboration, and systematic error analysis. Implementing measures like staff education, coordination, and computerization can reduce errors, enhancing efficiency and accuracy.

### Authors Contribution:

**AT:** Acquisition of data, manuscript writing, and analysis after review, all revisions and is responsible for the integrity and accuracy of research.

**MZ:** Conceived, prepared draft, designed, step by step guidance and did final review,

**ZR:** data collection, reviewing and suggestion in revising the manuscript.

**FZT:** Review after the manuscript was finalized.
